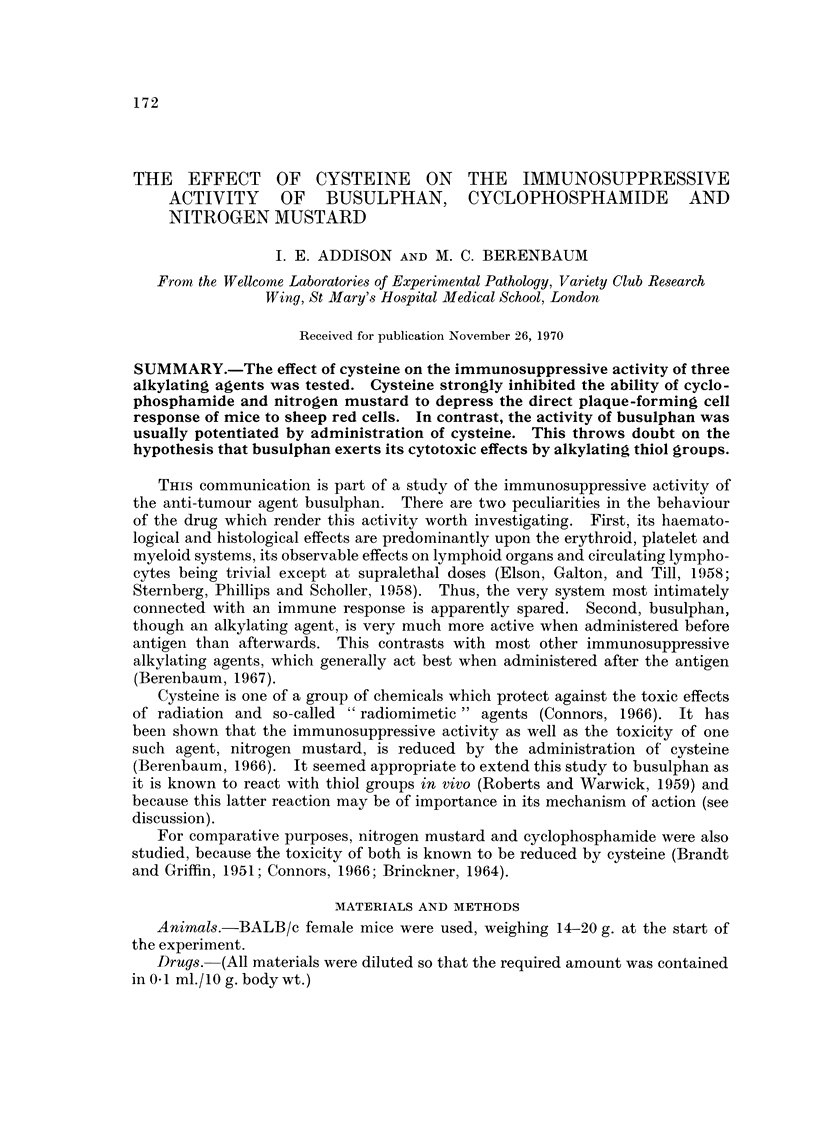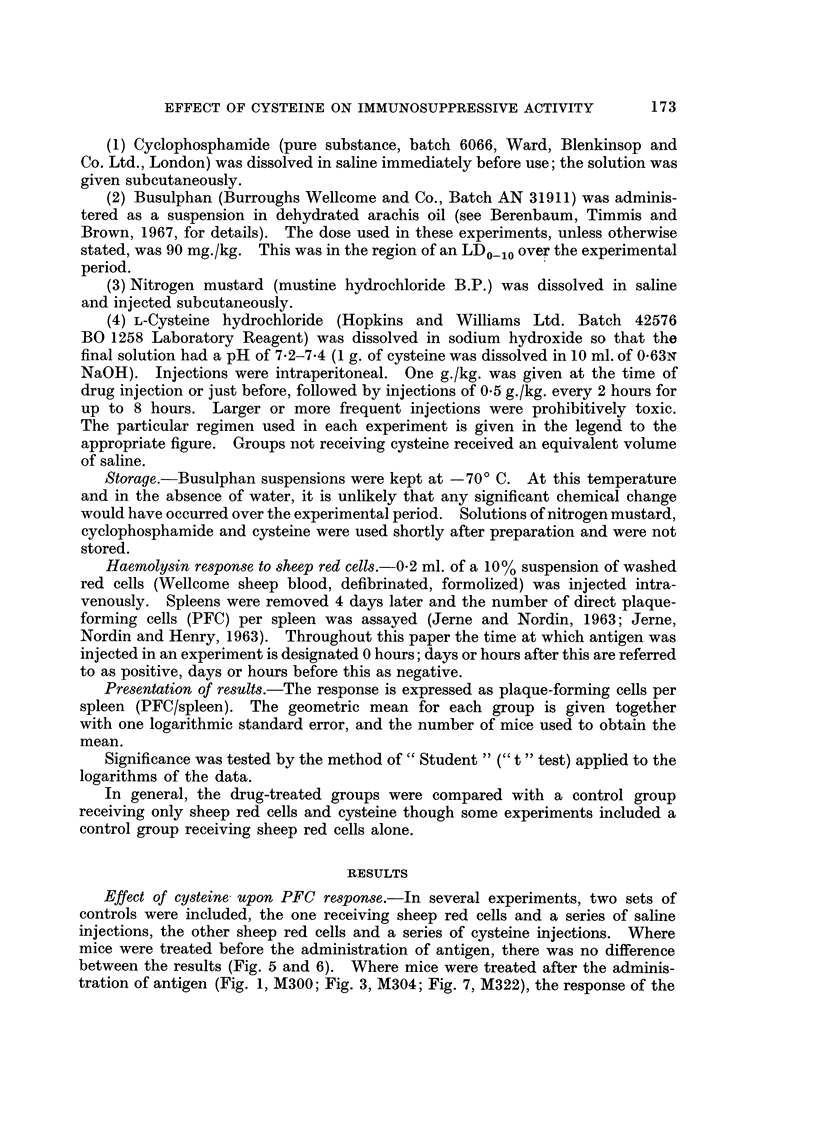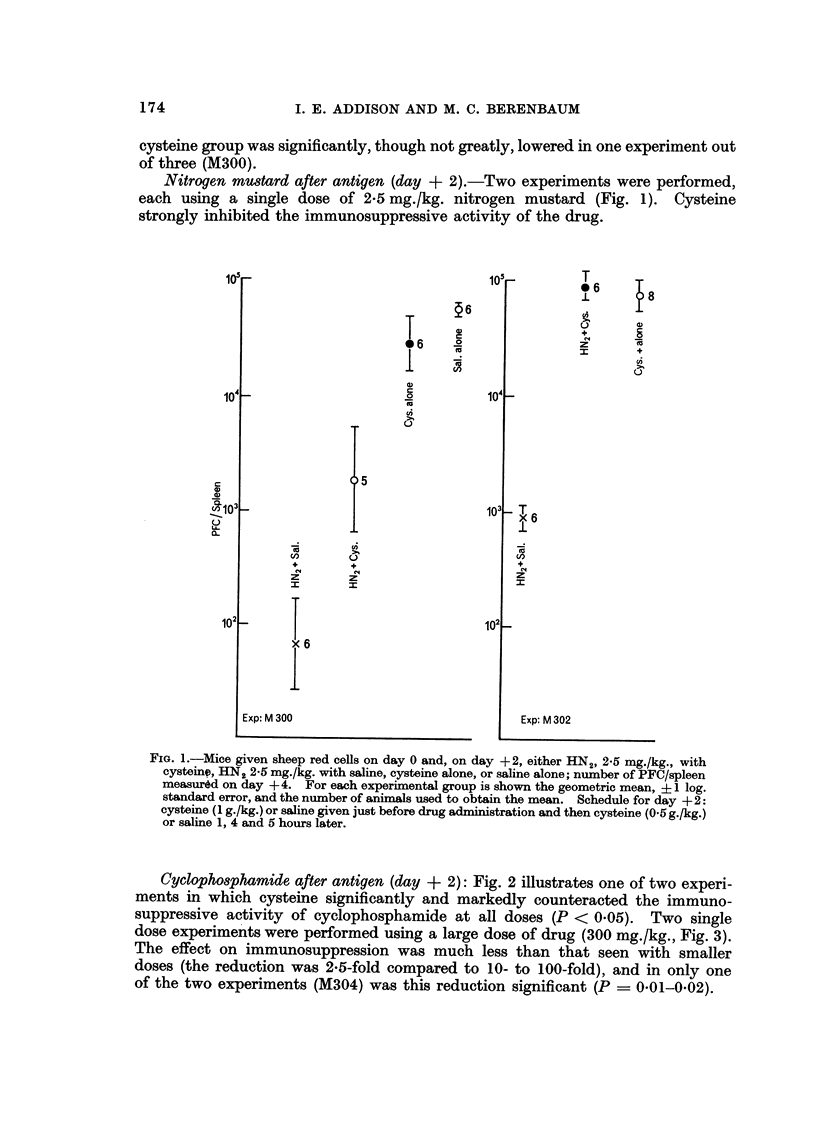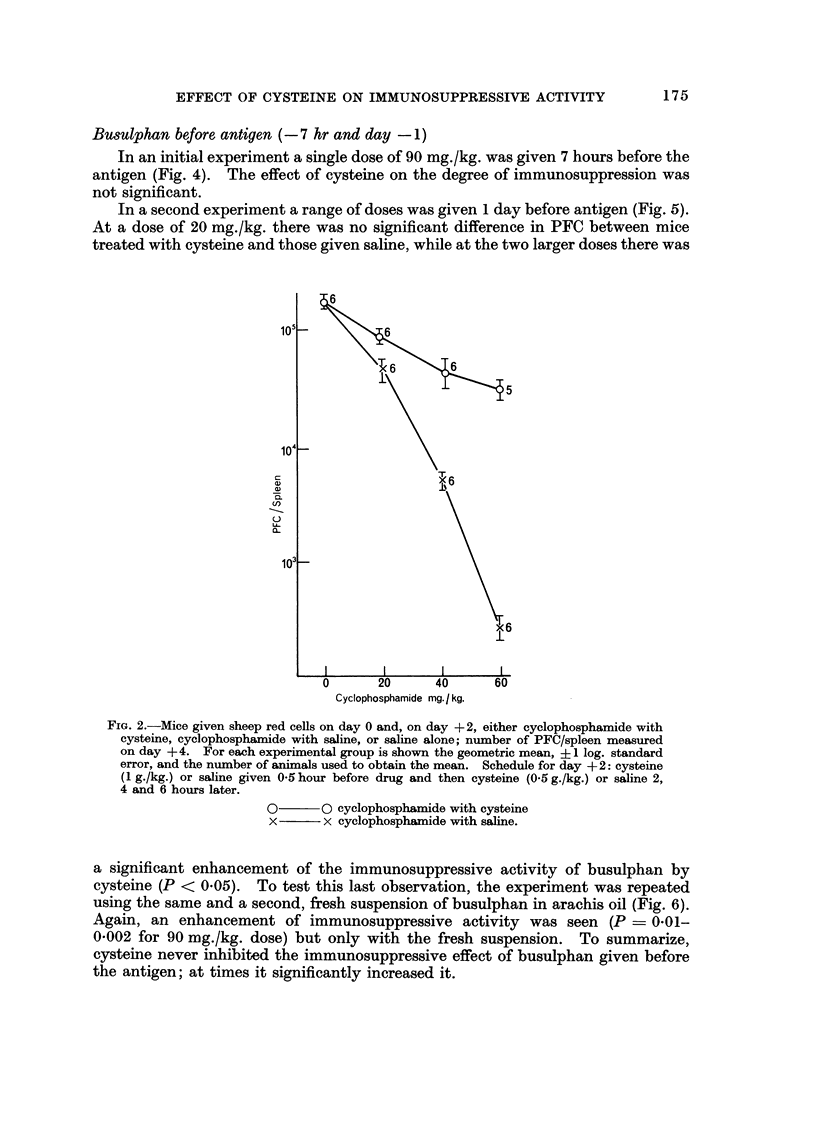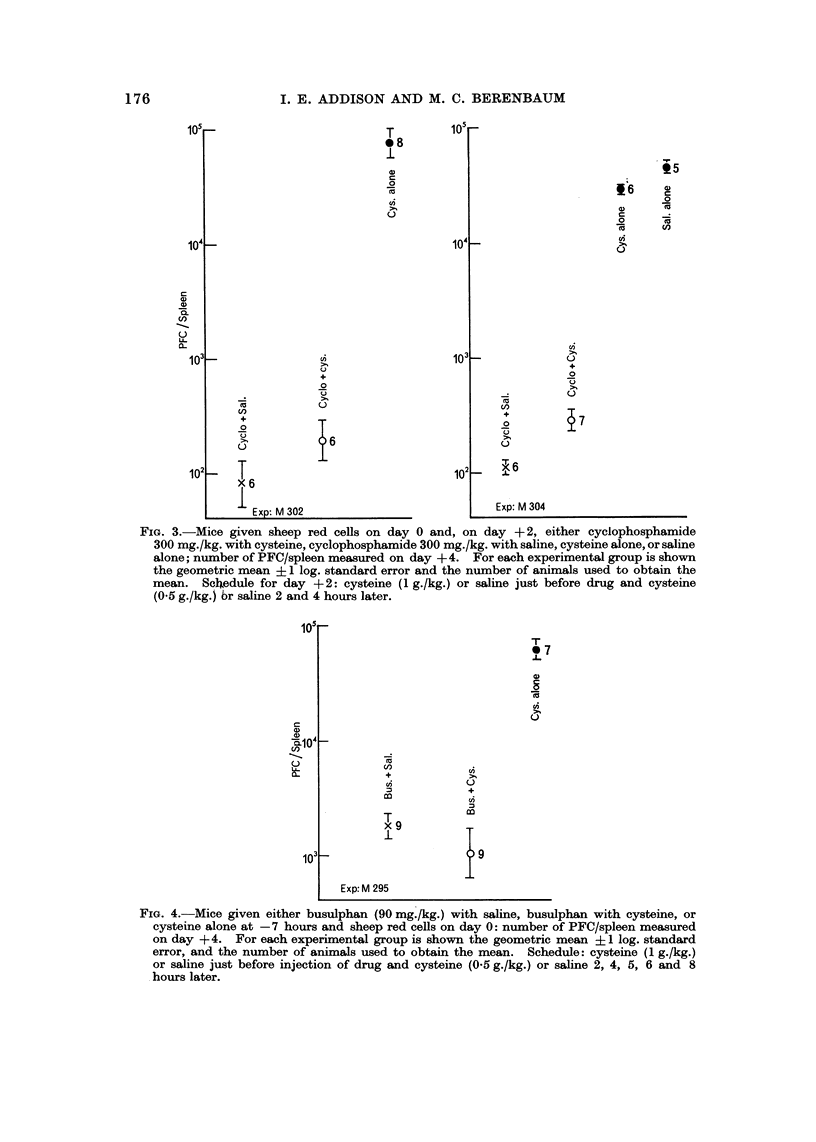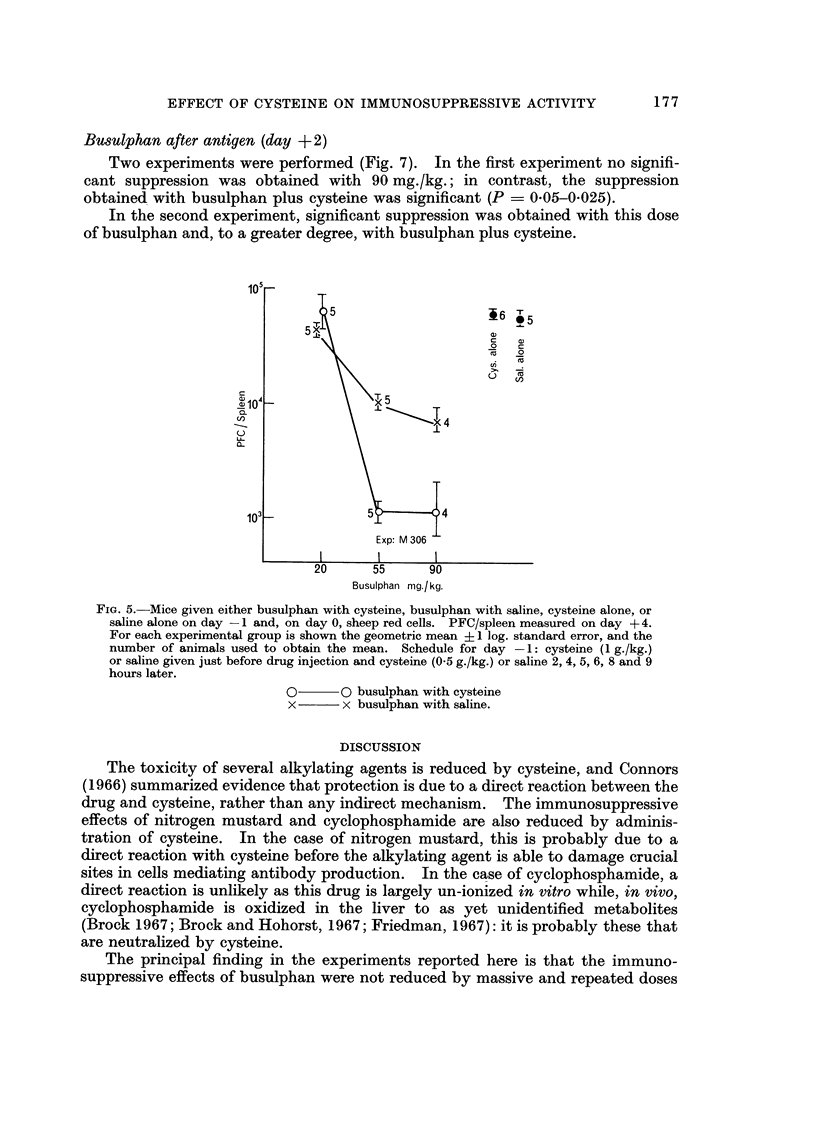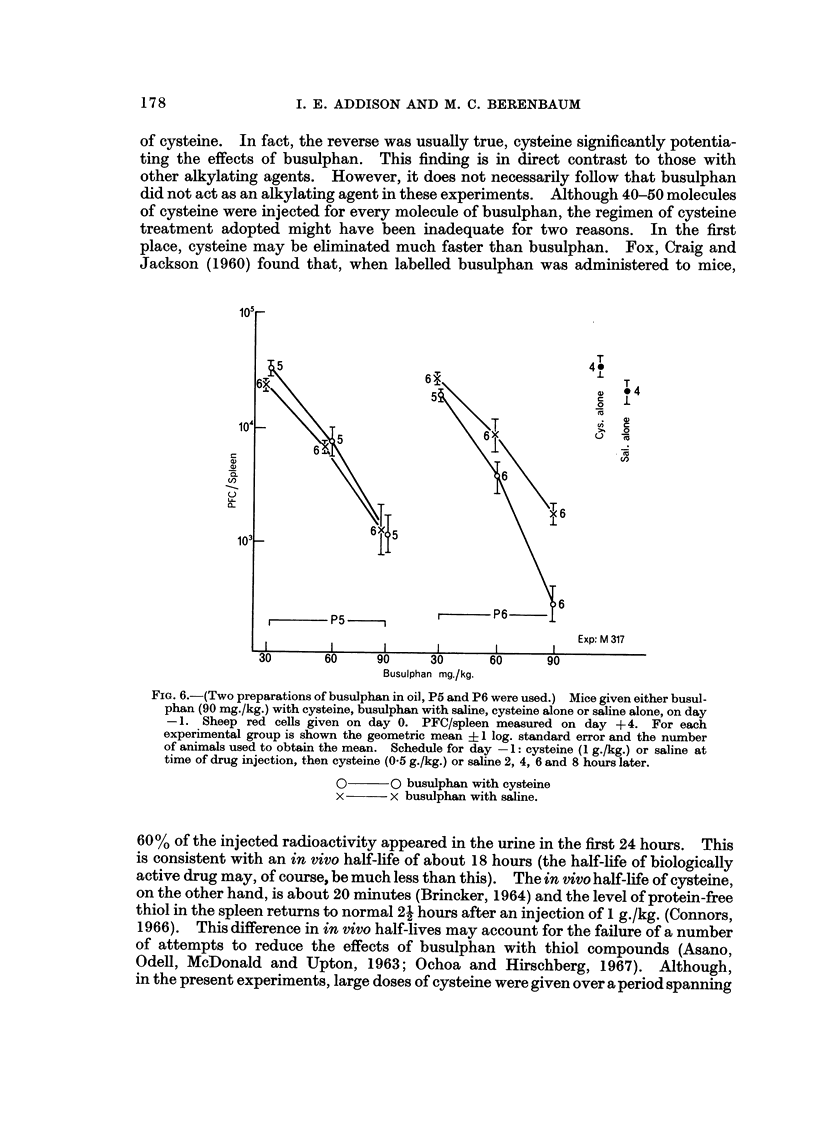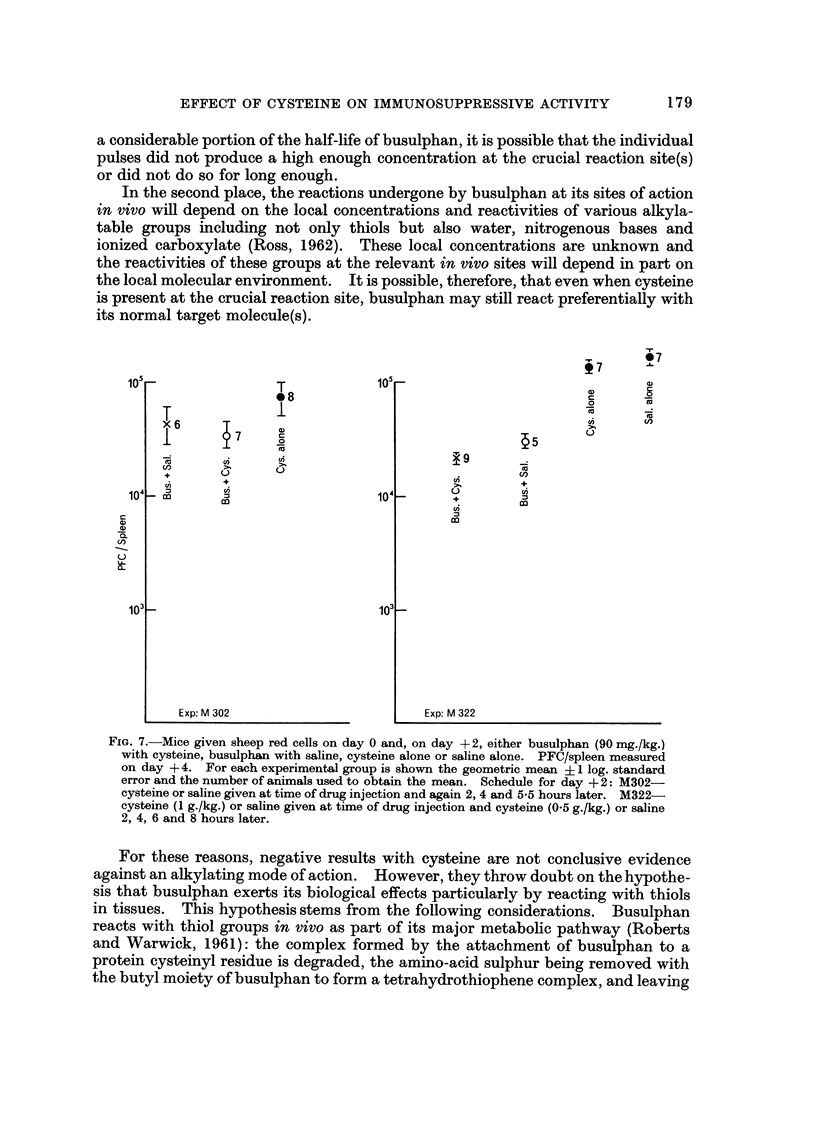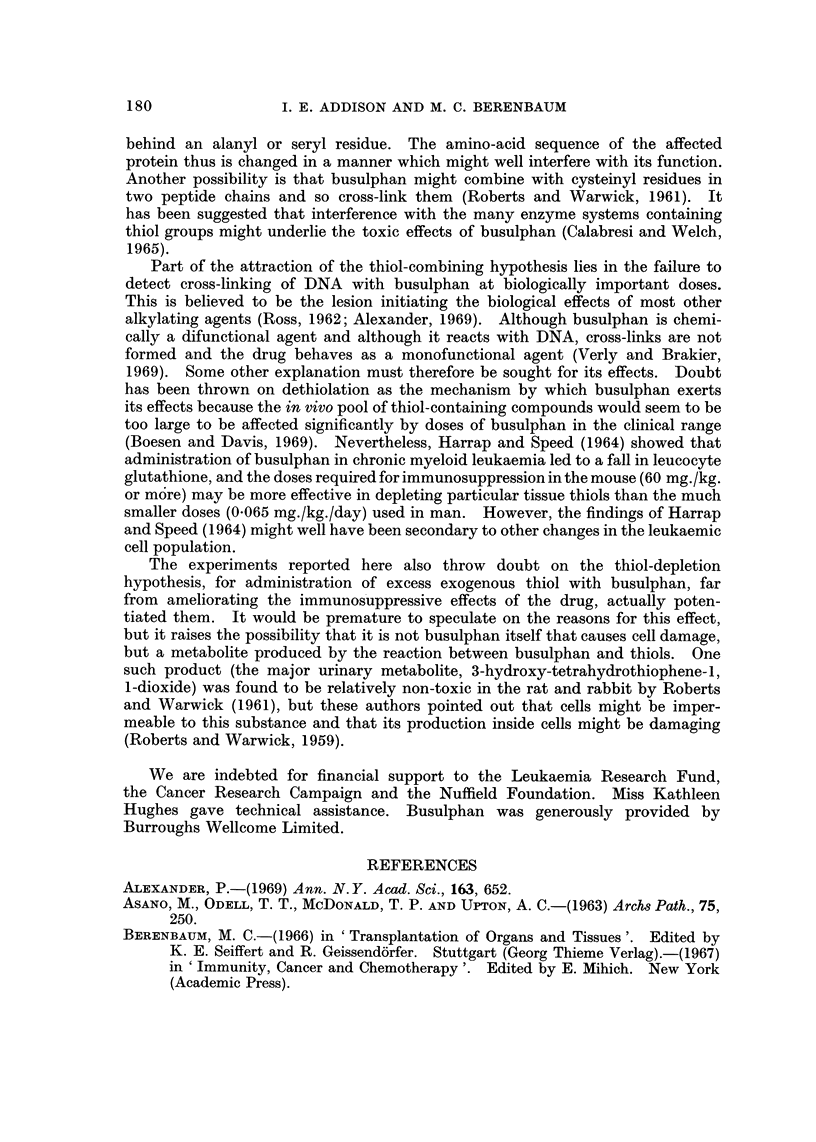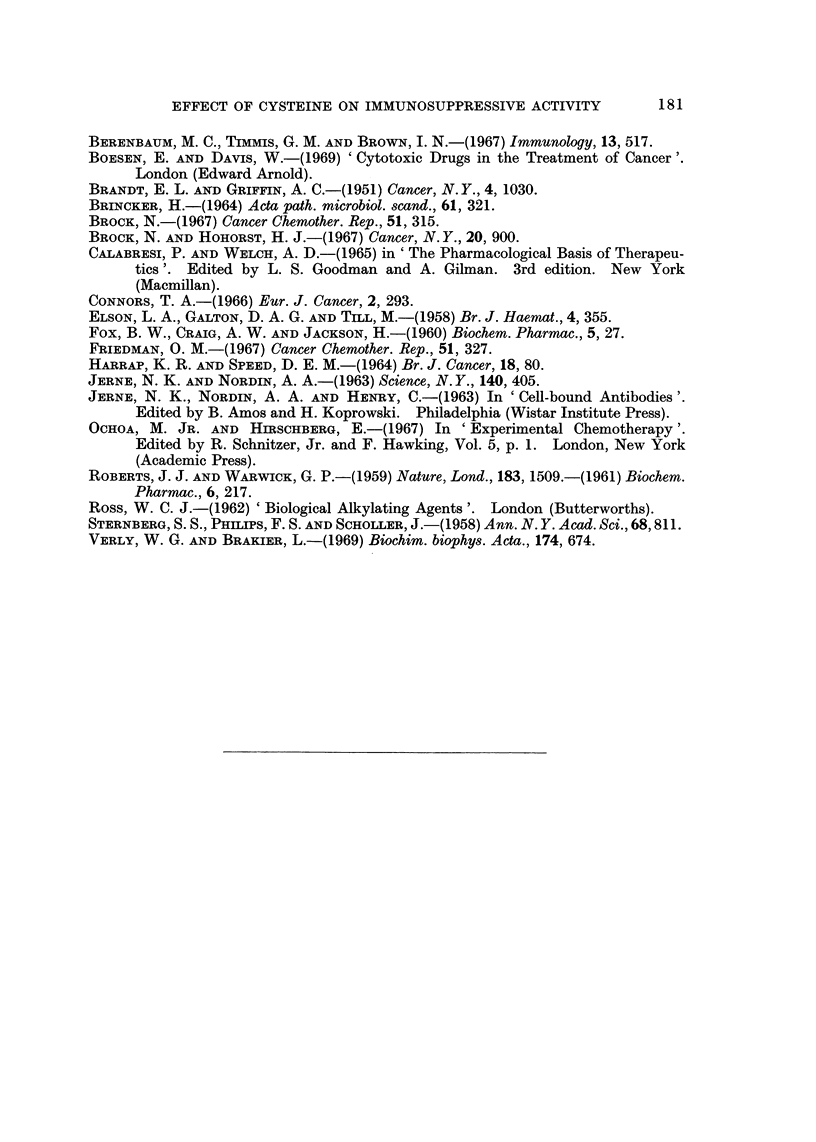# The Effect of Cysteine on the Immunosuppressive Activity of Busulphan, Cyclophosphamide and Nitrogen Mustard

**DOI:** 10.1038/bjc.1971.24

**Published:** 1971-03

**Authors:** I. E. Addison, M. C. Berenbaum

## Abstract

The effect of cysteine on the immunosuppressive activity of three alkylating agents was tested. Cysteine strongly inhibited the ability of cyclophosphamide and nitrogen mustard to depress the direct plaque-forming cell response of mice to sheep red cells. In contrast, the activity of busulphan was usually potentiated by administration of cysteine. This throws doubt on the hypothesis that busulphan exerts its cytotoxic effects by alkylating thiol groups.


### THE EFFECT OF CYSTEINE ON THE IMMUNOSUPPRESSIVE ACTIVITY OF BUSULPHAN, CYCLOPHOSPHAMIDE AND NITROGEN MUSTARD

I. E. ADDISON AND M. C. BERENBAUM

*From the Wellcome Laboratories of Experimental Pathology, Variety Club Research Wing, St Mary's Hospital Medical School, London*

Received for publication November 26, 1970

**SUMMARY.—The effect of cysteine on the immunosuppressive activity of three alkylating agents was tested. Cysteine strongly inhibited the ability of cyclophosphamide and nitrogen mustard to depress the direct plaque-forming cell response of mice to sheep red cells. In contrast, the activity of busulphan was usually potentiated by administration of cysteine. This throws doubt on the hypothesis that busulphan exerts its cytotoxic effects by alkylating thiol groups.**

THIS communication is part of a study of the immunosuppressive activity of the anti-tumour agent busulphan. There are two peculiarities in the behaviour of the drug which render this activity worth investigating. First, its haematological and histological effects are predominantly upon the erythroid, platelet and myeloid systems, its observable effects on lymphoid organs and circulating lymphocytes being trivial except at supralethal doses (Elson, Galton, and Till, 1958; Sternberg, Phillips and Scholler, 1958). Thus, the very system most intimately connected with an immune response is apparently spared. Second, busulphan, though an alkylating agent, is very much more active when administered before antigen than afterwards. This contrasts with most other immunosuppressive alkylating agents, which generally act best when administered after the antigen (Berenbaum, 1967).

Cysteine is one of a group of chemicals which protect against the toxic effects of radiation and so-called "radiomimetic" agents (Connors, 1966). It has been shown that the immunosuppressive activity as well as the toxicity of one such agent, nitrogen mustard, is reduced by the administration of cysteine (Berenbaum, 1966). It seemed appropriate to extend this study to busulphan as it is known to react with thiol groups *in vivo* (Roberts and Warwick, 1959) and because this latter reaction may be of importance in its mechanism of action (see discussion).

For comparative purposes, nitrogen mustard and cyclophosphamide were also studied, because the toxicity of both is known to be reduced by cysteine (Brandt and Griffin, 1951; Connors, 1966; Brinckner, 1964).

### MATERIALS AND METHODS

*Animals.*—BALB/c female mice were used, weighing 14–20 g. at the start of the experiment.

*Drugs.*—(All materials were diluted so that the required amount was contained in 0·1 ml./10 g. body wt.)

(1) Cyclophosphamide (pure substance, batch 6066, Ward, Blenkinsop and Co. Ltd., London) was dissolved in saline immediately before use; the solution was given subcutaneously.

(2) Busulphan (Burroughs Wellcome and Co., Batch AN 31911) was administered as a suspension in dehydrated arachis oil (see Berenbaum, Timmis and Brown, 1967, for details). The dose used in these experiments, unless otherwise stated, was 90 mg./kg. This was in the region of an $LD_{0-10}$ over the experimental period.

(3) Nitrogen mustard (mustine hydrochloride B.P.) was dissolved in saline and injected subcutaneously.

(4) L-Cysteine hydrochloride (Hopkins and Williams Ltd. Batch 42576 BO 1258 Laboratory Reagent) was dissolved in sodium hydroxide so that the final solution had a pH of 7·2–7·4 (1 g. of cysteine was dissolved in 10 ml. of 0·63N NaOH). Injections were intraperitoneal. One g./kg. was given at the time of drug injection or just before, followed by injections of 0·5 g./kg. every 2 hours for up to 8 hours. Larger or more frequent injections were prohibitively toxic. The particular regimen used in each experiment is given in the legend to the appropriate figure. Groups not receiving cysteine received an equivalent volume of saline.

*Storage.*—Busulphan suspensions were kept at −70° C. At this temperature and in the absence of water, it is unlikely that any significant chemical change would have occurred over the experimental period. Solutions of nitrogen mustard, cyclophosphamide and cysteine were used shortly after preparation and were not stored.

*Haemolysin response to sheep red cells.*—0·2 ml. of a 10% suspension of washed red cells (Wellcome sheep blood, defibrinated, formolized) was injected intravenously. Spleens were removed 4 days later and the number of direct plaque-forming cells (PFC) per spleen was assayed (Jerne and Nordin, 1963; Jerne, Nordin and Henry, 1963). Throughout this paper the time at which antigen was injected in an experiment is designated 0 hours; days or hours after this are referred to as positive, days or hours before this as negative.

*Presentation of results.*—The response is expressed as plaque-forming cells per spleen (PFC/spleen). The geometric mean for each group is given together with one logarithmic standard error, and the number of mice used to obtain the mean.

Significance was tested by the method of " Student " (" t " test) applied to the logarithms of the data.

In general, the drug-treated groups were compared with a control group receiving only sheep red cells and cysteine though some experiments included a control group receiving sheep red cells alone.

### RESULTS

*Effect of cysteine upon PFC response.*—In several experiments, two sets of controls were included, the one receiving sheep red cells and a series of saline injections, the other sheep red cells and a series of cysteine injections. Where mice were treated before the administration of antigen, there was no difference between the results (Fig. 5 and 6). Where mice were treated after the administration of antigen (Fig. 1, M300; Fig. 3, M304; Fig. 7, M322), the response of the cysteine group was significantly, though not greatly, lowered in one experiment out of three (M300).

*Nitrogen mustard after antigen* (*day* + 2).—Two experiments were performed, each using a single dose of 2·5 mg./kg. nitrogen mustard (Fig. 1). Cysteine strongly inhibited the immunosuppressive activity of the drug.

FIG. 1.—Mice given sheep red cells on day 0 and, on day +2, either $HN_2$, 2·5 mg./kg., with cysteine, $HN_2$ 2·5 mg./kg. with saline, cysteine alone, or saline alone; number of PFC/spleen measured on day +4. For each experimental group is shown the geometric mean, ±1 log. standard error, and the number of animals used to obtain the mean. Schedule for day +2: cysteine (1 g./kg.) or saline given just before drug administration and then cysteine (0·5 g./kg.) or saline 1, 4 and 5 hours later.

*Cyclophosphamide after antigen* (*day* + 2): Fig. 2 illustrates one of two experiments in which cysteine significantly and markedly counteracted the immunosuppressive activity of cyclophosphamide at all doses ($P < 0{\cdot}05$). Two single dose experiments were performed using a large dose of drug (300 mg./kg., Fig. 3). The effect on immunosuppression was much less than that seen with smaller doses (the reduction was 2·5-fold compared to 10- to 100-fold), and in only one of the two experiments (M304) was this reduction significant ($P = 0{\cdot}01$–$0{\cdot}02$).

*Busulphan before antigen (—7 hr and day —1)*

In an initial experiment a single dose of 90 mg./kg. was given 7 hours before the antigen (Fig. 4). The effect of cysteine on the degree of immunosuppression was not significant.

In a second experiment a range of doses was given 1 day before antigen (Fig. 5). At a dose of 20 mg./kg. there was no significant difference in PFC between mice treated with cysteine and those given saline, while at the two larger doses there was

FIG. 2.—Mice given sheep red cells on day 0 and, on day +2, either cyclophosphamide with cysteine, cyclophosphamide with saline, or saline alone; number of PFC/spleen measured on day +4. For each experimental group is shown the geometric mean, ±1 log. standard error, and the number of animals used to obtain the mean. Schedule for day +2: cysteine (1 g./kg.) or saline given 0·5 hour before drug and then cysteine (0·5 g./kg.) or saline 2, 4 and 6 hours later.

O——O cyclophosphamide with cysteine
×——× cyclophosphamide with saline.

a significant enhancement of the immunosuppressive activity of busulphan by cysteine ($P < 0{\cdot}05$). To test this last observation, the experiment was repeated using the same and a second, fresh suspension of busulphan in arachis oil (Fig. 6). Again, an enhancement of immunosuppressive activity was seen ($P = 0{\cdot}01$–$0{\cdot}002$ for 90 mg./kg. dose) but only with the fresh suspension. To summarize, cysteine never inhibited the immunosuppressive effect of busulphan given before the antigen; at times it significantly increased it.

Fig. 3.—Mice given sheep red cells on day 0 and, on day +2, either cyclophosphamide 300 mg./kg. with cysteine, cyclophosphamide 300 mg./kg. with saline, cysteine alone, or saline alone; number of PFC/spleen measured on day +4. For each experimental group is shown the geometric mean ±1 log. standard error and the number of animals used to obtain the mean. Schedule for day +2: cysteine (1 g./kg.) or saline just before drug and cysteine (0·5 g./kg.) or saline 2 and 4 hours later.

Fig. 4.—Mice given either busulphan (90 mg./kg.) with saline, busulphan with cysteine, or cysteine alone at −7 hours and sheep red cells on day 0: number of PFC/spleen measured on day +4. For each experimental group is shown the geometric mean ±1 log. standard error, and the number of animals used to obtain the mean. Schedule: cysteine (1 g./kg.) or saline just before injection of drug and cysteine (0·5 g./kg.) or saline 2, 4, 5, 6 and 8 hours later.

*Busulphan after antigen* (*day* +2)

Two experiments were performed (Fig. 7). In the first experiment no significant suppression was obtained with 90 mg./kg.; in contrast, the suppression obtained with busulphan plus cysteine was significant ($P$ = 0·05–0·025).

In the second experiment, significant suppression was obtained with this dose of busulphan and, to a greater degree, with busulphan plus cysteine.

FIG. 5.—Mice given either busulphan with cysteine, busulphan with saline, cysteine alone, or saline alone on day −1 and, on day 0, sheep red cells. PFC/spleen measured on day +4. For each experimental group is shown the geometric mean ±1 log. standard error, and the number of animals used to obtain the mean. Schedule for day −1: cysteine (1 g./kg.) or saline given just before drug injection and cysteine (0·5 g./kg.) or saline 2, 4, 5, 6, 8 and 9 hours later.

O———O busulphan with cysteine
X———X busulphan with saline.

### DISCUSSION

The toxicity of several alkylating agents is reduced by cysteine, and Connors (1966) summarized evidence that protection is due to a direct reaction between the drug and cysteine, rather than any indirect mechanism. The immunosuppressive effects of nitrogen mustard and cyclophosphamide are also reduced by administration of cysteine. In the case of nitrogen mustard, this is probably due to a direct reaction with cysteine before the alkylating agent is able to damage crucial sites in cells mediating antibody production. In the case of cyclophosphamide, a direct reaction is unlikely as this drug is largely un-ionized *in vitro* while, *in vivo*, cyclophosphamide is oxidized in the liver to as yet unidentified metabolites (Brock 1967; Brock and Hohorst, 1967; Friedman, 1967): it is probably these that are neutralized by cysteine.

The principal finding in the experiments reported here is that the immunosuppressive effects of busulphan were not reduced by massive and repeated doses of cysteine. In fact, the reverse was usually true, cysteine significantly potentiating the effects of busulphan. This finding is in direct contrast to those with other alkylating agents. However, it does not necessarily follow that busulphan did not act as an alkylating agent in these experiments. Although 40–50 molecules of cysteine were injected for every molecule of busulphan, the regimen of cysteine treatment adopted might have been inadequate for two reasons. In the first place, cysteine may be eliminated much faster than busulphan. Fox, Craig and Jackson (1960) found that, when labelled busulphan was administered to mice,

FIG. 6.—(Two preparations of busulphan in oil, P5 and P6 were used.) Mice given either busulphan (90 mg./kg.) with cysteine, busulphan with saline, cysteine alone or saline alone, on day −1. Sheep red cells given on day 0. PFC/spleen measured on day +4. For each experimental group is shown the geometric mean ±1 log. standard error and the number of animals used to obtain the mean. Schedule for day −1: cysteine (1 g./kg.) or saline at time of drug injection, then cysteine (0·5 g./kg.) or saline 2, 4, 6 and 8 hours later.

O——O busulphan with cysteine
×——× busulphan with saline.

60% of the injected radioactivity appeared in the urine in the first 24 hours. This is consistent with an *in vivo* half-life of about 18 hours (the half-life of biologically active drug may, of course, be much less than this). The *in vivo* half-life of cysteine, on the other hand, is about 20 minutes (Brincker, 1964) and the level of protein-free thiol in the spleen returns to normal 2¼ hours after an injection of 1 g./kg. (Connors, 1966). This difference in *in vivo* half-lives may account for the failure of a number of attempts to reduce the effects of busulphan with thiol compounds (Asano, Odell, McDonald and Upton, 1963; Ochoa and Hirschberg, 1967). Although, in the present experiments, large doses of cysteine were given over a period spanning a considerable portion of the half-life of busulphan, it is possible that the individual pulses did not produce a high enough concentration at the crucial reaction site(s) or did not do so for long enough.

In the second place, the reactions undergone by busulphan at its sites of action *in vivo* will depend on the local concentrations and reactivities of various alkylatable groups including not only thiols but also water, nitrogenous bases and ionized carboxylate (Ross, 1962). These local concentrations are unknown and the reactivities of these groups at the relevant *in vivo* sites will depend in part on the local molecular environment. It is possible, therefore, that even when cysteine is present at the crucial reaction site, busulphan may still react preferentially with its normal target molecule(s).

FIG. 7.—Mice given sheep red cells on day 0 and, on day +2, either busulphan (90 mg./kg.) with cysteine, busulphan with saline, cysteine alone or saline alone. PFC/spleen measured on day +4. For each experimental group is shown the geometric mean $\pm 1$ log. standard error and the number of animals used to obtain the mean. Schedule for day +2: M302—cysteine or saline given at time of drug injection and again 2, 4 and 5·5 hours later. M322—cysteine (1 g./kg.) or saline given at time of drug injection and cysteine (0·5 g./kg.) or saline 2, 4, 6 and 8 hours later.

For these reasons, negative results with cysteine are not conclusive evidence against an alkylating mode of action. However, they throw doubt on the hypothesis that busulphan exerts its biological effects particularly by reacting with thiols in tissues. This hypothesis stems from the following considerations. Busulphan reacts with thiol groups *in vivo* as part of its major metabolic pathway (Roberts and Warwick, 1961): the complex formed by the attachment of busulphan to a protein cysteinyl residue is degraded, the amino-acid sulphur being removed with the butyl moiety of busulphan to form a tetrahydrothiophene complex, and leaving behind an alanyl or seryl residue. The amino-acid sequence of the affected protein thus is changed in a manner which might well interfere with its function. Another possibility is that busulphan might combine with cysteinyl residues in two peptide chains and so cross-link them (Roberts and Warwick, 1961). It has been suggested that interference with the many enzyme systems containing thiol groups might underlie the toxic effects of busulphan (Calabresi and Welch, 1965).

Part of the attraction of the thiol-combining hypothesis lies in the failure to detect cross-linking of DNA with busulphan at biologically important doses. This is believed to be the lesion initiating the biological effects of most other alkylating agents (Ross, 1962; Alexander, 1969). Although busulphan is chemically a difunctional agent and although it reacts with DNA, cross-links are not formed and the drug behaves as a monofunctional agent (Verly and Brakier, 1969). Some other explanation must therefore be sought for its effects. Doubt has been thrown on dethiolation as the mechanism by which busulphan exerts its effects because the *in vivo* pool of thiol-containing compounds would seem to be too large to be affected significantly by doses of busulphan in the clinical range (Boesen and Davis, 1969). Nevertheless, Harrap and Speed (1964) showed that administration of busulphan in chronic myeloid leukaemia led to a fall in leucocyte glutathione, and the doses required for immunosuppression in the mouse (60 mg./kg. or more) may be more effective in depleting particular tissue thiols than the much smaller doses (0·065 mg./kg./day) used in man. However, the findings of Harrap and Speed (1964) might well have been secondary to other changes in the leukaemic cell population.

The experiments reported here also throw doubt on the thiol-depletion hypothesis, for administration of excess exogenous thiol with busulphan, far from ameliorating the immunosuppressive effects of the drug, actually potentiated them. It would be premature to speculate on the reasons for this effect, but it raises the possibility that it is not busulphan itself that causes cell damage, but a metabolite produced by the reaction between busulphan and thiols. One such product (the major urinary metabolite, 3-hydroxy-tetrahydrothiophene-1, 1-dioxide) was found to be relatively non-toxic in the rat and rabbit by Roberts and Warwick (1961), but these authors pointed out that cells might be impermeable to this substance and that its production inside cells might be damaging (Roberts and Warwick, 1959).

We are indebted for financial support to the Leukaemia Research Fund, the Cancer Research Campaign and the Nuffield Foundation. Miss Kathleen Hughes gave technical assistance. Busulphan was generously provided by Burroughs Wellcome Limited.

### REFERENCES

ALEXANDER, P.—(1969) *Ann. N.Y. Acad. Sci.*, **163**, 652.

ASANO, M., ODELL, T. T., McDONALD, T. P. AND UPTON, A. C.—(1963) *Archs Path.*, **75**, 250.

BERENBAUM, M. C.—(1966) in ‘ Transplantation of Organs and Tissues ’. Edited by K. E. Seiffert and R. Geissendörfer. Stuttgart (Georg Thieme Verlag).—(1967) in ‘ Immunity, Cancer and Chemotherapy ’. Edited by E. Mihich. New York (Academic Press).

BERENBAUM, M. C., TIMMIS, G. M. AND BROWN, I. N.—(1967) *Immunology*, **13**, 517.
BOESEN, E. AND DAVIS, W.—(1969) 'Cytotoxic Drugs in the Treatment of Cancer'. London (Edward Arnold).
BRANDT, E. L. AND GRIFFIN, A. C.—(1951) *Cancer, N.Y.*, **4**, 1030.
BRINCKER, H.—(1964) *Acta path. microbiol. scand.*, **61**, 321.
BROCK, N.—(1967) *Cancer Chemother. Rep.*, **51**, 315.
BROCK, N. AND HOHORST, H. J.—(1967) *Cancer, N.Y.*, **20**, 900.
CALABRESI, P. AND WELCH, A. D.—(1965) in 'The Pharmacological Basis of Therapeutics'. Edited by L. S. Goodman and A. Gilman. 3rd edition. New York (Macmillan).
CONNORS, T. A.—(1966) *Eur. J. Cancer*, **2**, 293.
ELSON, L. A., GALTON, D. A. G. AND TILL, M.—(1958) *Br. J. Haemat.*, **4**, 355.
FOX, B. W., CRAIG, A. W. AND JACKSON, H.—(1960) *Biochem. Pharmac.*, **5**, 27.
FRIEDMAN, O. M.—(1967) *Cancer Chemother. Rep.*, **51**, 327.
HARRAP, K. R. AND SPEED, D. E. M.—(1964) *Br. J. Cancer*, **18**, 80.
JERNE, N. K. AND NORDIN, A. A.—(1963) *Science, N.Y.*, **140**, 405.
JERNE, N. K., NORDIN, A. A. AND HENRY, C.—(1963) In 'Cell-bound Antibodies'. Edited by B. Amos and H. Koprowski. Philadelphia (Wistar Institute Press).
OCHOA, M. JR. AND HIRSCHBERG, E.—(1967) In 'Experimental Chemotherapy'. Edited by R. Schnitzer, Jr. and F. Hawking, Vol. 5, p. 1. London, New York (Academic Press).
ROBERTS, J. J. AND WARWICK, G. P.—(1959) *Nature, Lond.*, **183**, 1509.—(1961) *Biochem. Pharmac.*, **6**, 217.
ROSS, W. C. J.—(1962) 'Biological Alkylating Agents'. London (Butterworths).
STERNBERG, S. S., PHILIPS, F. S. AND SCHOLLER, J.—(1958) *Ann. N.Y. Acad. Sci.*, **68**, 811.
VERLY, W. G. AND BRAKIER, L.—(1969) *Biochim. biophys. Acta.*, **174**, 674.